# Elotuzumab for the treatment of extramedullary myeloma: a retrospective analysis of clinical efficacy and SLAMF7 expression patterns

**DOI:** 10.1007/s00277-021-04447-6

**Published:** 2021-02-11

**Authors:** Sophia Danhof, Leo Rasche, Anja Mottok, Tabea Steinmüller, Xiang Zhou, Martin Schreder, Teresa Kilian, Susanne Strifler, Andreas Rosenwald, Michael Hudecek, Hermann Einsele, Elena Gerhard-Hartmann

**Affiliations:** 1 Department of Internal Medicine II, University Hospital Würzburg, Würzburg, Germany; 2 Mildred Scheel Early Career Center Würzburg, Würzburg, Germany; 3 Institute of Human Genetics, Ulm University and University Medical Center, Ulm, Germany; 4 Department of Pathology, University of Würzburg, Würzburg, Germany; 5 First Department of Medicine, Center for Oncology and Hematology, Klinik Ottakring, Vienna, Austria

**Keywords:** Plasma cells, CD319, CS1, Monoclonal antibody, Extramedullary disease, Antigen loss

## Abstract

Extramedullary disease (EMD) represents a high-risk state of multiple myeloma (MM) associated with poor prognosis. While most anti-myeloma therapeutics demonstrate limited efficacy in this setting, some studies exploring the utility of chimeric antigen receptor (CAR)-modified T cells reported promising results. We have recently designed SLAMF7-directed CAR T cells for the treatment of MM. SLAMF7 is a transmembrane receptor expressed on myeloma cells that plays a role in myeloma cell homing to the bone marrow. Currently, the only approved anti-SLAMF7 therapeutic is the monoclonal antibody elotuzumab, but its efficacy in EMD has not been investigated thoroughly. Thus, we retrospectively analyzed the efficacy of elotuzumab-based combination therapy in a cohort of 15 patients with EMD. Moreover, since the presence of the target antigen is an indispensable prerequisite for effective targeted therapy, we investigated the SLAMF7 expression on extramedullary located tumor cells before and after treatment. We observed limited efficacy of elotuzumab-based combination therapies, with an overall response rate of 40% and a progression-free and overall survival of 3.8 and 12.9 months, respectively. Before treatment initiation, all available EMD tissue specimens (*n* = 3) demonstrated a strong and consistent SLAMF7 surface expression by immunohistochemistry. Furthermore, to investigate a potential antigen reduction under therapeutic selection pressure, we analyzed samples of de novo EMD (*n* = 3) outgrown during elotuzumab treatment. Again, immunohistochemistry documented strong and consistent SLAMF7 expression in all samples. In aggregate, our data point towards a retained expression of SLAMF7 in EMD and encourage the development of more potent SLAMF7-directed immunotherapies, such as CAR T cells.

## Introduction

Multiple myeloma (MM) is a malignant plasma cell dyscrasia that accounts for approximately 10% of all hematological malignancies. Albeit primarily a disease of the bone marrow, extramedullary myeloma manifestations occur in up to 37% of patients [28], and incidence increases over the course of the disease [3]. It has been postulated that myeloma cells must undergo relevant alterations of the adhesion, migration, and/or chemokine receptor profile [34] in order to lose their strong dependence on the bone marrow microenvironment and to obtain capacities required for extramedullary spread. In patients with extramedullary disease (EMD), high-risk cytogenetics are overrepresented [1, 4, 9, 31]. Furthermore, an association of altered expression of specific non-coding RNA molecules and EMD has been reported [2, 18]. However, the exact molecular mechanisms underlying the development of EMD remain largely unclear. Clinical studies consistently demonstrate an inferior prognosis for patients with EMD, with overall survival periods of less than 12 months in the relapsed/refractory setting in some cohorts [16, 40, 42]. In non-randomized treatment series, even regimens containing highly potent novel agents such as carfilzomib [42], pomalidomide [20], and daratumumab [39] achieve response rates of less than 30% in this context. Despite the urgent medical need, randomized clinical studies explicitly investigating the treatment of EMD are yet to be performed.

Based on the promising results of the ELOQUENT-2 trial, elotuzumab was the first monoclonal antibody that gained FDA/EMA approval [11] for patients with relapsed/refractory MM (RRMM) in combination with dexamethasone and lenalidomide. Subsequently, the ELOQUENT-3 trial [10] reported encouraging data for elotuzumab in combination with pomalidomide and dexamethasone in heavily pretreated patients with an overall response rate (ORR) of 53% and a median progression-free survival (PFS) of 10.3 months, leading to the additional approval of this combination therapy. Regrettably, although patients with EMD were included in the approval studies, detailed treatment response and outcome data for this subgroup have so far not been reported. Elotuzumab targets the signaling lymphocytic activation molecule (SLAM) family member 7 (SLAMF7, aliases: CD319, CS1, CRACC), an immunomodulatory transmembrane receptor that is highly and uniformly expressed on malignant plasma cells [19], natural killer cells, and other immune cells [6, 15]. SLAMF7 is a homotypic receptor that plays a role for myeloma cell homing to the bone marrow niche [38]. However, the downstream signaling network induced upon SLAMF7 activation in myeloma cells-and thus the functional relevance of SLAMF7 expression for the maintenance of the disease-remains unclear to date [17].

As in other tumor entities, the concept of chimeric antigen receptor (CAR)-modified T cells opens up novel treatment options for patients suffering from MM. CAR-modified T cells targeting the B-cell maturation antigen (BCMA, CD269) have recently demonstrated promising efficacy in high-risk RRMM [33], including deep responses in patients with substantial extraosseous disease [5, 7, 29, 35]. However, the emergence of antigen loss variants during therapy with BCMA-directed CAR T cells has been documented, leading to fulminant recurrence of the disease [5]. Interestingly, antigen reduction under therapeutic selection pressure has so far not been reported for SLAMF7 [36]. Thus, **w**e have generated a SLAMF7-directed CAR equipped with an antigen-binding domain derived from elotuzumab [15]. As SLAMF7 mediates myeloma cell adherence to the bone marrow, we hypothesized that SLAMF7 expression could be reduced in EMD and hence limit the efficacy of SLAMF7-directed therapy. Therefore, we retrospectively analyzed the efficacy of elotuzumab-based therapy, as well as expression levels of SLAMF7 in a small series of patients with RRMM and EMD. We show that elotuzumab had limited efficacy for the treatment of EMD; however, this was not due to reduced SLAMF7 expression levels as those were comparably high and consistent in all tissue specimens investigated. In our view, these data encourage the further development of SLAMF7-directed CAR T cells for the treatment of high-risk MM and phase I clinical evaluation is currently ongoing (NCT04499339).

## Patients and methods

### Patients

We performed a retrospective analysis at our tertiary care hospital of all patients with RRMM and EMD receiving an elotuzumab-based combination therapy from January 2016 to January 2020. We defined RRMM based on the current consensus recommendations of the International Myeloma Working Group (IMWG) [30]. Evidence of infiltration with aberrant plasma cells in tissue biopsies from extraosseous sites led to the diagnosis of EMD. All histopathological diagnoses were performed by expert hematopathologists. Radiological evaluation (CT, PET/CT, MRI) was used for the diagnosis of EMD, if tissue sampling was impossible. Retrospective analysis of clinical data and evaluation of SLAMF7 expression on myeloma samples were approved by the ethical committee of the Medical Faculty of the University of Wuerzburg, Germany (reference number: 61/18).

### Assessment of response and outcome

We performed response assessment according to the IMWG Response Criteria [22]. According to the Kaplan–Meier method, PFS was calculated from initiation of elotuzumab-based treatment to confirmed serological or EMD progression or death attributed to disease progression, whichever occurred first; overall survival (OS) was calculated from initiation of elotuzumab-based treatment to death from any cause. The remaining patients were censored at the last date of follow-up. For statistical analyses, we used the Prism Software 7.0 (GraphPad, San Diego, CA).

### Immunohistochemistry

Immunohistochemistry for SLAMF7 and CD138 was performed on formalin-fixed paraffin-embedded tissue slides (HPA055945/Sigma-Aldrich, dilution 1:1000 and MI15/DAKO, dilution 1:100, respectively) using the MEDAC HiDef Detection™ HRP Polymer System and DAB-2V (Histofine) according to the manufacturers’ instructions and standard immunohistochemical protocols. Normal tonsil specimens were used as control tissue.

## Results

### Patient characteristics

From January 2016 to January 2020, we treated a total of 74 RRMM patients with an elotuzumab-based combination therapy, 15 (21%) of whom showed EMD. Only the latter were included for further analysis (Table 1). Within this collective, patients were diagnosed with MM at a median age of 62 (range 32-80) years and received elotuzumab-based therapy at a median of 4.5 (0.8-19) years after initial diagnosis. Approximately half of the patients were female (*n* = 8; 53%), the majority of the patients had initially presented with advanced disease (Salmon&Durie stage III, *n* = 12; 80%), and one-third of the patients had high-risk cytogenetics (*n* = 5; 33%) [27]. In half of the patients (*n* = 8; 53%), all detectable EMD lesions were adjacent to bone, while a minority had only EMD without any bone-adjacent lesions (*n* = 3; 20%) and the rest of the patients presented with a mixed picture (*n* = 4; 27%). Most frequent localizations of EMD manifestations were paravertebral (*n* = 12; 80%), soft tissues without adjacency to bone (*n* = 4; 27%), parenchymal organs, and lymph nodes (both *n* = 3; 20%). Prior to elotuzumab treatment, the patients had received a median of four (range 1-9) therapy lines. A majority of patients had previously undergone high-dose chemotherapy followed by autologous hematopoietic stem cell transplantation (*n* = 11; 73%). All patients had received treatment with bortezomib, and half of them had received treatment with a next-generation proteasome inhibitor (*n* = 8; 53%). All but one patient had been exposed to lenalidomide (*n* = 14; 93%), and half of them had additionally been exposed to one (*n* = 5; 33%) or two (*n* = 2, 13%) alternative immunomodulatory drugs (IMiDs). Almost half of the patients were refractory to their last line of therapy (*n* = 7; 47%).

**Table 1:** Clinical patient characteristics

Patients, *n* (%)	15 (100)
Age at dx, median (range)	62 (32-80)
Subtype, *n* (%)	
IgG	11 (73)
IgA	2 (13)
LC	2 (13)
Salmon&Durie	
I	2 (13)
II	1 (7)
IIIA	10 (67)
IIIB	2 (13)
Sex, *n* (%)	
Female	8 (53)
Male	7 (47)
Cytogenetics, *n* (%)	
High risk*	5 (33)
Standard risk	7 (47)
NA	3 (20)
Start of elotuzumab, median time from dx in months (range)	54 (10-228)
Prior lines of therapy, *n* (%)	
1-3	7 (47)
4-6	5 (33)
> 6	3 (20)
Prior treatment, *n* (%)	
Stem cell transplantation	11 (73)
Autologous	11 (73)
Allogeneic	3 (20)
Proteasome inhibitors	15 (100)
Bortezomib	15 (100)
Carfilzomib	7 (47)
Ixazomib	1 (7)
Immunomodulatory drugs	14 (93)
Thalidomide	3 (20)
Lenalidomide	14 (93)
Pomalidomide	6 (40)
Daratumumab	7 (47)
Localization of EMD	
Paravertebral	12 (80)
Soft tissue without adjacency to bone	4 (27)
Parenchymal organ	3 (20)
Lymph nodes	3 (20)
Pleura	2 (13)
Skin	1 (7)

*dx* diagnosis; *Ig* immunoglobulin; *LC* light chain; *NA* not applicable; *EMD* extramedullary disease

*High risk as defined by the presence of del(17p) and/or t(4;14) and/or t(14;16) [27]

### Treatment protocol

Two 4-week cycles of weekly elotuzumab applications (10 mg/kg body weight (BW) i.v.) were followed by fortnightly elotuzumab infusions (10 mg/kg BW) in combination with lenalidomide (*n* = 5; 33%), or monthly elotuzumab infusions (20 mg/kg BW) in combination with pomalidomide (*n* = 10; 67%). The IMiDs were administered orally throughout the first 3 weeks of each cycle at doses according to the treating physician’s choice. Dexamethasone was administered once weekly (20-40 mg). Treatment was continued until progression.

### Response to therapy

In this cohort, patients received a median number of three (range 1-17) treatment cycles. Upon evaluation of the best serological response, the ORR was 40%, with one patient (7%) achieving a very good partial response and five patients (33%) achieving a partial response (PR). In five other patients (33%), we observed temporary stabilization of disease, while four patients (27%) were refractory to the elotuzumab-based therapeutic regimen.

Follow-up imaging was available for two-thirds of patients (*n* = 10; 67%). Regression or stable disease of the extramedullary lesions was noted in four patients (27%). Progressive EMD was observed in six patients (40%). Radiological and serological evaluation of response was consistent in most patients (*n* = 8; 53%). Two patients (13%) demonstrated better disease control on imaging than on serological evaluation: One individual achieved complete regression of the extramedullary lesion despite serological PR and another individual maintained PR on imaging despite continuous serological progression. At time of relapse, two patients (13%) experienced extramedullary progression despite ongoing serological response.

**Fig. 1:**
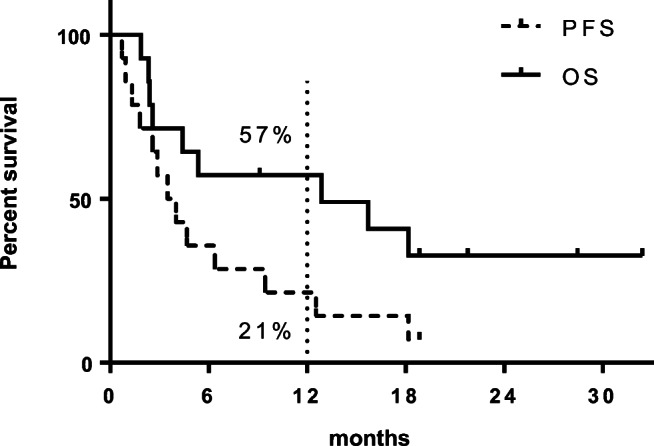
Progression-free survival (PFS, dashed line) and overall survival (OS, continuous line) of patients (*n* = 15). One-year PFS and OS are visualized by the dotted line

Upon survival analysis, the median PFS and OS in this cohort were 3.8 and 12.9 months, respectively (Fig. 1). The 1-year PFS and OS rates were 21% and 57%, respectively. Due to the limited number of patients in this study, we did not perform additional subgroup analyses.

### Expression of SLAMF7 on extramedullary myeloma cells prior to elotuzumab treatment

**Fig. 2:**
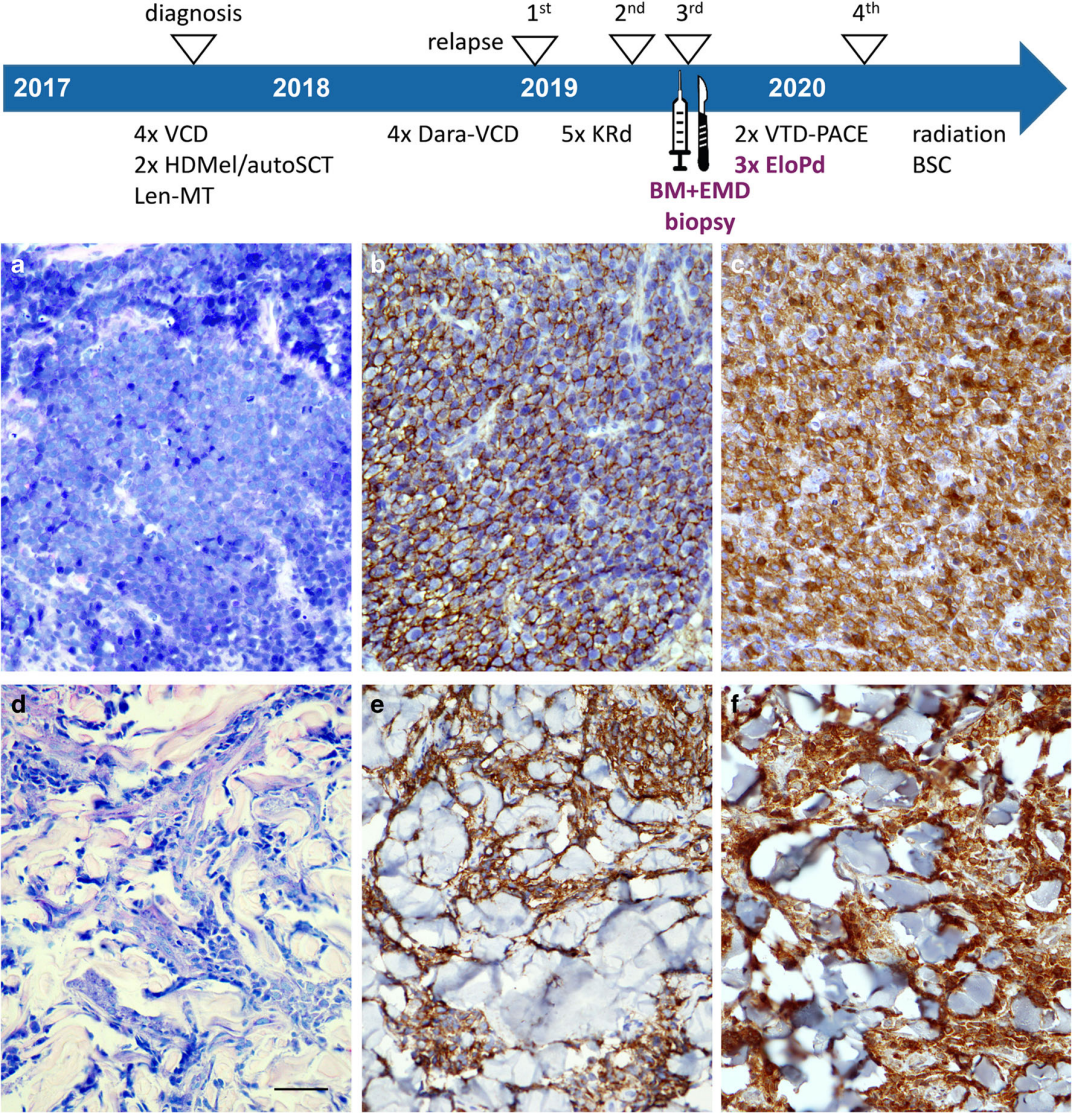
SLAMF7 expression on intramedullary and extramedullary myeloma cells prior to elotuzumab treatment. Giemsa (**a**, **d**) and immunohistochemical staining for CD138 (**b**, **e**) as well as SLAMF7 (**c**, **f**) in the bone marrow (**a**-**c**) and in a skin biopsy (**d**-**f**) obtained simultaneously prior to elotuzumab treatment (**a**-**f**: original magnification ×400; length of the scale bar in **d**: 50 μm). SLAMF7 shows a strong and consistent expression in the CD138-positive myeloma cells. *VCD*, bortezomib/cyclophosphamide/dexamethasone; *HD-Mel/autoSCT*, high-dose melphalan/autologous stem cell transplantation; *Len-MT*, lenalidomide maintenance; *Dara*, daratumumab; *KRd*, carfilzomib/lenalidomide/dexamethasone; *BM*, bone marrow; *EMD*, extramedullary disease; *VTD-PACE*, bortezomib/thalidomide/dexamethasone-cisplatin/adriamycin/cyclophosphamide/etoposide); *EloPd*, elotuzumab/pomalidomide/dexamethasone; *BSC*, best supportive care

In search of reasons for the relatively poor response and outcome rates in the investigated patient collective, we considered reduced expression of SLAMF7 on the myeloma cells at extramedullary sites as a potential mechanism for limited elotuzumab efficacy. Thus, we aimed to investigate SLAMF7 protein expression on myeloma cells at extramedullary sites. We queried our database for patients who had undergone biopsies of EMD before initiation of elotuzumab treatment and identified a small number of patients (*n* = 3; 20%) for whom the EMD material was available in our tissue biobank. We further retrieved matching bone marrow biopsies from the respective individuals prior to elotuzumab therapy. Immunohistochemical stainings to evaluate SLAMF7 expression in CD138+ myeloma cells (Figs 2) documented strong and consistent SLAMF7 expression in all EMD and bone marrow biopsies (Table 2), indicating a preserved expression of the antigen in myeloma cells even when located outside the bone marrow niche. Interestingly, the clinical course under elotuzumab treatment was highly variable in these patients and PFS ranged from 1.3 to 9.4 months (Table 2).

**Table 2:** Clinical and immunohistochemical profile of patients with histologically confirmed extramedullary myeloma

Identifier		E1	E3^#1^	E8	E16	E17	E18^#2^
General	Age at dx	47	68	46	60	54	55
	Sex	m	m	m	m	m	m
	Subtype	λLC	IgGκ	IgGλ	IgGκ	IgAκ	IgGκ
	S&D at dx	IIIB	IA	IIIA	IIIA	IIIB	IIIA
	ISS at dx	2	1	ND	2	3	ND
	FISH	Normal	t(4;14), +1q21	ND	del13q14, del1p32, +1q21	del17p, t(4;14), del13q14, +1q21	Normal
	Previous lines of therapy	3	3	4	5	3	2
	Prior SCT, type (number)	Auto (2)	Auto (2)	Auto (1) allo (1)	Auto (2)	Auto (2)	Auto (3)
	Prior IMiDs	Len	Thal, Len	Len	Len, Pom	Len, Pom	None
	Prior PIs	Bort	Bort, Carf	Bort, Carf	Bort	Bort	Bort
	Prior mAbs	None	Dara	Dara	None	None	None
	EMD prior to elotuzumab	Yes	Yes	Yes	No	No	No
Elotuzumab treatment	Time from dx (months)	26	27	144	41	15	186
	Combination IMiD	Pom	Pom	Pom	Pom	Len, Pom	Len
	Treatment cycles	10	3	1	1	3	7
	Best response	PR	PR	PD	SD	SD	PR
	PFS (months)	9.4	2.9	1.3	0.9	2.7	9.0
	OS (months)	12.9	4.4	5.4	1.0	4.7	>9.8^#3^
	Type of progression	Serological + EMD	Serological + EMD	EMD	EMD	EMD	Serological + EMD
IHC	Localization of EMD	Paravertebral soft tissue	Skin	Paravertebral soft tissue	Maxillary mucosa	Lymph node	Thoracic wall
	SLAMF7 on CD138+ EM MM	+++^#4^	+++	+++	+++	+++	+++
	SLAMF7 on CD138+ BM MM	+++	+++	+++	+++	+++	+++

*dx* diagnosis; *S&D* Salmon&Durie; *LC* light chain; *Ig* immunoglobulin; *ISS* International Staging System; *ND* not done; *FISH* fluorescence in situ hybridization; *SCT* stem cell transplantation; *auto* autologous; *allo* allogeneic; *IMiD* immunomodulatory drug; *Len* lenalidomide; *Thal* thalidomide; *Pom* pomalidomide; *PI* proteasome inhibitor; *Bort* bortezomib; *Carf* carfilzomib; *mAb* monoclonal antibody; *Dara* daratumumab; *EMD* extramedullary disease; *Elo* elotuzumab; *PR* partial response; *PD* progressive disease; *SD* stable disease; *PFS* progression-free survival; *OS* overall survival; *IHC* immunohistochemistry; *EM* extramedullary; *MM* multiple myeloma; *BM* bone marrow

^#1^Fig. 2

^#2^Fig. 3

^#3^Lost to follow-up

^#4^Strong and consistent expression

### Expression of SLAMF7 on de novo extramedullary myeloma during elotuzumab treatment

**Fig. 3:**
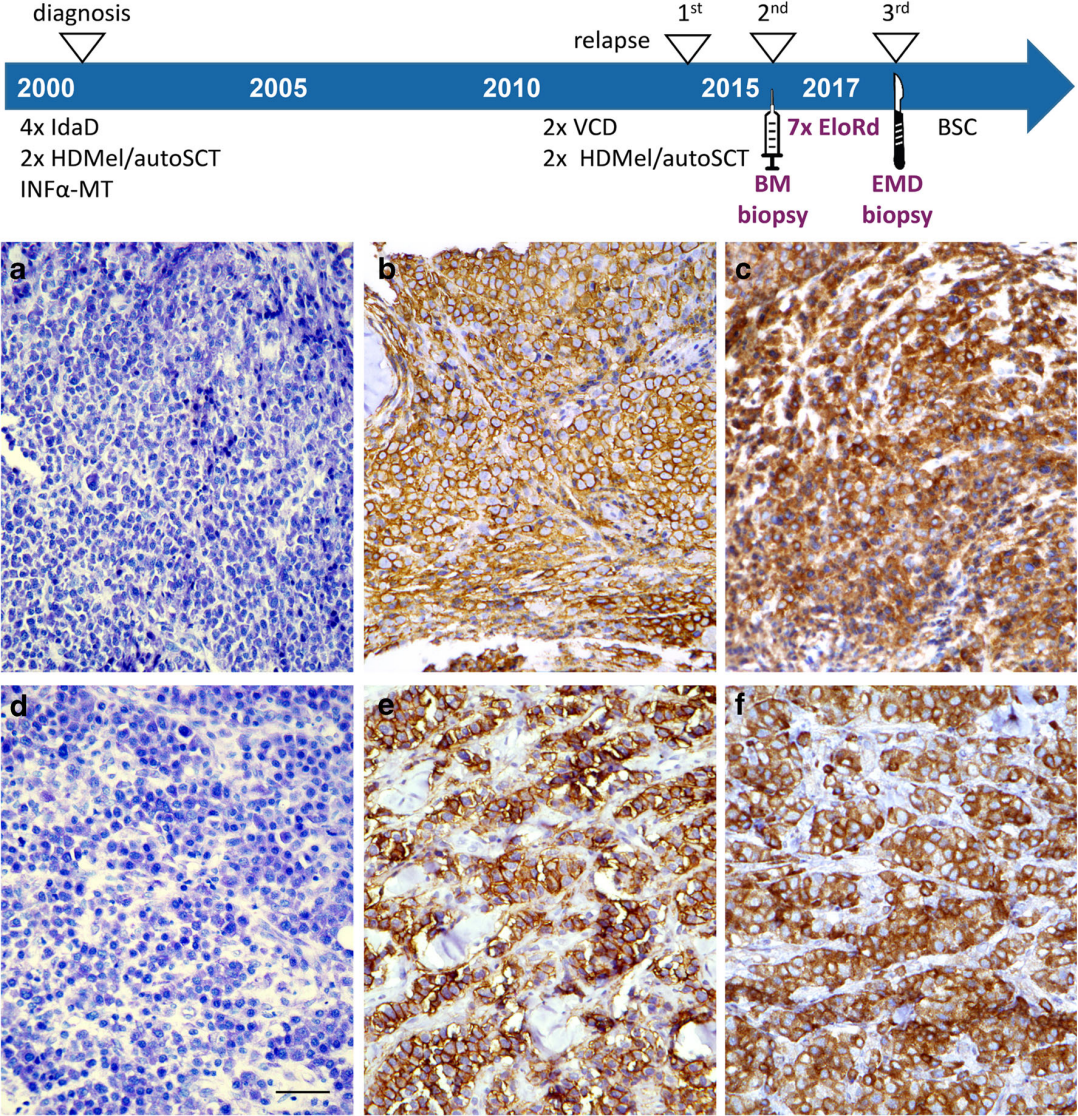
SLAMF7 expression on bone marrow myeloma cells prior to and on extramedullary myeloma cells after elotuzumab treatment. Giemsa (**a**, **d**) and immunohistochemical staining for CD138 (**b**, **e**) as well as SLAMF7 (**c**, **f**) in the bone marrow (**a**-**c**) and a biopsy from the chest wall (**d**-**f**) (**a**-**f**: original magnification ×400; length of the scale bar in **d**: 50 μm). The former was obtained prior to elotuzumab treatment, and the latter was obtained 9 months later, after 7 cycles of elotuzumab-based combination therapy. SLAMF7 shows a strong and consistent expression in the CD138-positive myeloma cells. *IdaD*, idarubicin/dexamethasone; *HD-Mel/autoSCT*, high-dose melphalan/autologous stem cell transplantation; *IFNα-MT*, interferon α maintenance; *VCD*, bortezomib/cyclophosphamide/dexamethasone; *BM*, bone marrow; *EloRd*, elotuzumab/lenalidomide/dexamethasone; *EMD*, extramedullary disease; *BSC*, best supportive care

A subset of patients in our study cohort developed additional or progressive EMD manifestations during elotuzumab therapy, but tissue biopsies from those lesions were not available. Thus, to investigate the expression of SLAMF7 on myeloma cells of EMD under therapeutic selection pressure, we screened our electronic database for patients with de novo EMD during elotuzumab treatment. We identified five (out of 59) patients who were newly diagnosed with EMD after initiation of elotuzumab treatment, but before the start of the subsequent treatment line. For three patients, tissue specimens of EMD biopsies were available. As baseline controls, we identified bone marrow biopsies from the respective patients prior to elotuzumab therapy. When we compared immunohistochemical stainings of SLAMF7 on bone marrow myeloma cells before treatment and on extramedullary myeloma cells after treatment (Fig. 3), we found strong and consistent SLAMF7 expression in all cases. This indicated that outgrowth of SLAMF7^+^ extramedullary myeloma occurred despite SLAMF7-directed selection pressure. The time span from initiation of elotuzumab treatment to diagnosis of EMD ranged from 1 to 7 treatment cycles and PFS of the respective patients ranged from 0.9 to 9.0 months (Table 2). In aggregate, SLAMF7 was strongly and consistently expressed on all bone marrow and EMD specimens of MM, irrespective of previous exposition and clinical response to anti-SLAMF7 targeted immunotherapy with elotuzumab.

## Discussion

Monoclonal antibodies have become an important weapon in the therapeutic armamentarium against MM. However, their optimal incorporation into existing treatment paradigms remains to be defined, especially for patients with high-risk disease. To the best of our knowledge, this is the first study investigating the role of elotuzumab in EMD. While EMD patients were eligible for both elotuzumab approval trials [10, 23], EMD was removed as covariate from the regression analyses in the ELOQUENT-2 trial as consequence of reduced imaging interventions as per protocol amendment [23]. In ELOQUENT-3, lactate dehydrogenase (LDH) ≥ 300 U/l was used as surrogate parameter for EMD and subgroup analysis showed only moderate efficacy in this patient cohort with a hazard ratio of 0.75 (95% CI 0.31-1.84) [10]. However, Oka et al. reported a case of a patient with EMD resistant to lenalidomide/dexamethasone who responded to the addition of elotuzumab [26], whereas our group documented the occurrence of EMD during elotuzumab treatment in a small number of patients [8]. Taken together, the therapeutic value of elotuzumab for the treatment of EMD in patients with RRMM is unclear.

In the present study, the elotuzumab-based regimen demonstrated limited efficacy with an ORR of 40% and a moderate outcome with a 1-year PFS rate of 21%, respectively. ELOQUENT-2 and ELOQUENT-3, by contrast, had documented ORRs of 79% and 53%, and 1-year PFS rates of 68% and 43%, respectively [10, 23]. One possible explanation for the observed limited efficacy in the herein investigated patient cohort might be an overrepresentation of patients having undergone a high number of previous lines of therapy (median of 4 vs. 2 and 3, respectively), including intensive pretreatment regimens (prior stem cell transplantation in 73% in the present study vs. 52% in both ELOQUENT trials). Furthermore, in our patient cohort, previous exposure to IMiDs was considerably higher than in ELOQUENT-2 (exposure to lenalidomide in 93% vs. 5%) and in our study, more patients had high-risk cytogenetics than in ELOQUENT-3 (33% vs. 22%).

As the presence of the respective antigen is an obligatory prerequisite for efficient targeted therapy, we hypothesized that another reason for the observed inferior outcome data in our cohort could be reduced SLAMF7 expression on myeloma cells located outside the bone marrow niche. Previous analyses of SLAMF7 expression in MM focused mainly on phenotypic characterization of myeloma cells originating from the bone marrow; however, Hsi et al. [19] demonstrated mostly preserved SLAMF7 levels on myeloma cells in a small number of EMD biopsies. Here, we observed strong and consistent SLAMF7 protein expression determined by immunohistochemistry in all investigated tissue specimens, including six EMD biopsies. Considering that a functional relevance of SLAMF7 for the homing of myeloma cells to the bone marrow was postulated [38], our findings suggest that SLAMF7 does not imperatively restrict myeloma cells to the bone marrow microenvironment. Furthermore, we did not identify primary absence of the target antigen as reason for reduced efficacy of elotuzumab in extramedullary lesions.

Downregulation or reduction of target antigens under selection pressure is a common mechanism of resistance in cancer cells [12, 14, 21, 24]. In MM, this phenomenon has been observed for CD38 during daratumumab treatment [25] as well as for BCMA during CAR T cell therapy [5]. To the best of our knowledge, data on SLAMF7 negative myeloma cells has not been published. Indeed, in our small cohort, we did not detect reduced antigen expression despite extramedullary spread under the anti-SLAMF7 selection pressure of the elotuzumab treatment. This might point towards either a relatively weak therapeutic pressure of elotuzumab or a relevant role of SLAMF7 for the maintenance of the disease. Given that SLAMF7 is a self-ligand, we cannot exclude that for extramedullary spread of single cells, e.g. into peritoneum, pleural cavity, or cerebrospinal fluid, accompanied by a disintegration of myeloma cell aggregates, SLAMF7 might become more dispensable for the persistence of the disease. This is in line with a previously reported case of EMD, where the malignant pleural effusion harbored myeloma cells with lower SLAMF7 expression than the bone marrow [8]. However, the evaluation of subtle differences in SLAMF7 expression levels requires analysis by flow cytometry or other methodologies which outperform immunohistochemistry with regard to antigen quantification.

Other potential reasons for the here observed limited efficacy of elotuzumab in EMD could result from the biology of extramedullary myeloma cell growth. Myeloma cells that are capable of surviving outside the bone marrow have previously undergone a biological evolution, including chromosomal aberrations, mutations of individual genes, and epigenetic changes [3]. Furthermore, they demonstrate an increased proliferative index compared to bone marrow myeloma cells [13, 31]. Therefore, it is plausible that the therapeutic efficacy of elotuzumab may be limited by the overall more aggressive nature of the disease in this setting. Another important factor to be considered is difference in the microenvironment in bone marrow and extramedullary myeloma niche, but unfortunately, the immunological composition and interactions of EMD have been studied poorly to date. However, preclinical observations suggest that microenvironment-dependent inhibitory mechanisms might impair the efficacy of immunotherapy at extramedullary myeloma sites [37]. Also, the high incidence of EMD after allogeneic stem cell transplantation [28, 32] could be interpreted as spread to more immune-deprived sites.

We are aware of the limitations of the current study that are, in particular, related to its retrospective nature and the small sample size. However, we feel that such real-life data can provide valuable insights, especially for rare disease manifestations, and potentially guide the setup of prospective studies. In conclusion, our analyses demonstrate limited efficacy of current elotuzumab-based IMiD combination treatments in patients with EMD, despite strong and consistent presence of SLAMF7 on extramedullary myeloma cells. In our opinion, this observation encourages the further development of other potentially more potent SLAMF7-directed immunotherapies, such as antibody-drug conjugates [41] and CAR T cells [15].

## References

[1] Besse L, Sedlarikova L, Greslikova H, Kupska R, Almasi M, Penka M, Jelinek T, Pour L, Adam Z, Kuglik P, Krejci M, Hajek R, Sevcikova S (2016). Cytogenetics in multiple myeloma patients progressing into extramedullary disease. Eur J Haematol.

[2] Besse L, Sedlarikova L, Kryukov F, Nekvindova J, Radova L, Slaby O, Kuglik P, Almasi M, Penka M, Krejci M, Adam Z, Pour L, Sevcikova S, Hajek R (2015). Circulating serum microRNA-130a as a novel putative marker of extramedullary myeloma. PLoS One.

[3] Bhutani M, Foureau DM, Atrash S, Voorhees PM, Usmani SZ (2020). Extramedullary multiple myeloma. Leukemia.

[4] Billecke L, Murga Penas EM, May AM, Engelhardt M, Nagler A, Leiba M, Schiby G, Kröger N, Zustin J, Marx A, Matschke J, Tiemann M, Goekkurt E, Heidtmann HH, Vettorazzi E, Dierlamm J, Bokemeyer C, Schilling G (2013). Cytogenetics of extramedullary manifestations in multiple myeloma. Br J Haematol.

[5] Brudno JN, Maric I, Hartman SD, Rose JJ, Wang M, Lam N, Stetler-Stevenson M, Salem D, Yuan C, Pavletic S, Kanakry JA, Ali SA, Mikkilineni L, Feldman SA, Stroncek DF, Hansen BG, Lawrence J, Patel R, Hakim F, Gress RE, Kochenderfer JN (2018). T cells genetically modified to express an anti-B-cell maturation antigen chimeric antigen receptor cause remissions of poor-prognosis relapsed multiple myeloma. J Clin Oncol.

[6] Chen J, Zhong MC, Guo H, Davidson D, Mishel S, Lu Y, Rhee I, Pérez-Quintero LA, Zhang S, Cruz-Munoz ME, Wu N, Vinh DC, Sinha M, Calderon V, Lowell CA, Danska JS, Veillette A (2017). SLAMF7 is critical for phagocytosis of haematopoietic tumour cells via Mac-1 integrin. Nature.

[7] Cohen AD, Garfall AL, Stadtmauer EA, Melenhorst JJ, Lacey SF, Lancaster E, Vogl DT, Weiss BM, Dengel K, Nelson A, Plesa G, Chen F, Davis MM, Hwang WT, Young RM, Brogdon JL, Isaacs R, Pruteanu-Malinici I, Siegel DL, Levine BL, June CH, Milone MC (2019). B cell maturation antigen-specific CAR T cells are clinically active in multiple myeloma. J Clin Invest.

[8] Danhof S, Strifler S, Hose D, Kortüm M, Bittrich M, Hefner J, Einsele H, Knop S, Schreder M (2019). Clinical and biological characteristics of myeloma patients influence response to elotuzumab combination therapy. J Cancer Res Clin Oncol.

[9] Deng S, Xu Y, An G, Sui W, Zou D, Zhao Y, Qi J, Li F, Hao M, Qiu L (2015). Features of extramedullary disease of multiple myeloma: high frequency of p53 deletion and poor survival: a retrospective single-center study of 834 cases. Clin Lymphoma Myeloma Leuk.

[10] Dimopoulos MA, Dytfeld D, Grosicki S, Moreau P, Takezako N, Hori M, Leleu X, LeBlanc R, Suzuki K, Raab MS, Richardson PG, Popa McKiver M, Jou YM, Shelat SG, Robbins M, Rafferty B, San-Miguel J (2018). Elotuzumab plus pomalidomide and dexamethasone for multiple myeloma. N Engl J Med.

[11] Dimopoulos MA, Lonial S, Betts KA, Chen C, Zichlin ML, Brun A, Signorovitch JE, Makenbaeva D, Mekan S, Sy O, Weisel K, Richardson PG (2018). Elotuzumab plus lenalidomide and dexamethasone in relapsed/refractory multiple myeloma: extended 4-year follow-up and analysis of relative progression-free survival from the randomized ELOQUENT-2 trial. Cancer.

[12] Duffner U, Abdel-Mageed A, Younge J, Tornga C, Scott K, Staddon J, Elliott K, Stumph J, Kidd P (2016). The possible perils of targeted therapy. Leukemia.

[13] Firsova MV, Mendeleeva LP, Kovrigina AM, Solovev MV, Savchenko VG (2020). Plasmacytoma in patients with multiple myeloma: morphology and immunohistochemistry. BMC Cancer.

[14] Foran JM, Norton AJ, Micallef IN (2001). Loss of CD20 expression following treatment with rituximab (chimaeric monoclonal anti-CD20): a retrospective cohort analysis. Br J Haematol.

[15] Gogishvili T, Danhof S, Prommersberger S, Rydzek J, Schreder M, Brede C, Einsele H, Hudecek M (2017). SLAMF7-CAR T cells eliminate myeloma and confer selective fratricide of SLAMF7(+) normal lymphocytes. Blood.

[16] Gozzetti A, Cerase A, Lotti F, Rossi D, Palumbo A, Petrucci MT, Patriarca F, Nozzoli C, Cavo M, Offidani M, Floridia M, Berretta S, Vallone R, Musto P, Lauria F, Marchini E, Fabbri A, Oliva S, Zamagni E, Sapienza FG, Ballanti S, Mele G, Galli M, Pirrotta MT, di Raimondo F, GIMEMA (Gruppo Italiano Malattie Ematologiche dell'Adulto) Myeloma Working Party (2012). Extramedullary intracranial localization of multiple myeloma and treatment with novel agents: a retrospective survey of 50 patients. Cancer.

[17] Guo H, Cruz-Munoz ME, Wu N, Robbins M, Veillette A (2015). Immune cell inhibition by SLAMF7 is mediated by a mechanism requiring src kinases, CD45, and SHIP-1 that is defective in multiple myeloma cells. Mol Cell Biol.

[18] Handa H, Kuroda Y, Kimura K, Masuda Y, Hattori H, Alkebsi L, Matsumoto M, Kasamatsu T, Kobayashi N, Tahara KI, Takizawa M, Koiso H, Ishizaki T, Shimizu H, Yokohama A, Tsukamoto N, Saito T, Murakami H (2017). Long non-coding RNA MALAT1 is an inducible stress response gene associated with extramedullary spread and poor prognosis of multiple myeloma. Br J Haematol.

[19] Hsi ED, Steinle R, Balasa B, Szmania S, Draksharapu A, Shum BP, Huseni M, Powers D, Nanisetti A, Zhang Y, Rice AG, van Abbema A, Wong M, Liu G, Zhan F, Dillon M, Chen S, Rhodes S, Fuh F, Tsurushita N, Kumar S, Vexler V, Shaughnessy JD, Barlogie B, van Rhee F, Hussein M, Afar DEH, Williams MB (2008). CS1, a potential new therapeutic antibody target for the treatment of multiple myeloma. Clin Cancer Res.

[20] Jimenez-Segura R, Granell M, Gironella M (2019). Pomalidomide-dexamethasone for treatment of soft-tissue plasmacytomas in patients with relapsed/refractory multiple myeloma. Eur J Haematol.

[21] Kmieciak M, Knutson KL, Dumur CI (2007). HER-2/neu antigen loss and relapse of mammary carcinoma are actively induced by T cell-mediated anti-tumor immune responses. Eur J Immunol.

[22] Kumar S, Paiva B, Anderson KC, Durie B, Landgren O, Moreau P, Munshi N, Lonial S, Bladé J, Mateos MV, Dimopoulos M, Kastritis E, Boccadoro M, Orlowski R, Goldschmidt H, Spencer A, Hou J, Chng WJ, Usmani SZ, Zamagni E, Shimizu K, Jagannath S, Johnsen HE, Terpos E, Reiman A, Kyle RA, Sonneveld P, Richardson PG, McCarthy P, Ludwig H, Chen W, Cavo M, Harousseau JL, Lentzsch S, Hillengass J, Palumbo A, Orfao A, Rajkumar SV, Miguel JS, Avet-Loiseau H (2016). International Myeloma Working Group consensus criteria for response and minimal residual disease assessment in multiple myeloma. Lancet Oncol.

[23] Lonial S, Dimopoulos M, Palumbo A, White D, Grosicki S, Spicka I, Walter-Croneck A, Moreau P, Mateos MV, Magen H, Belch A, Reece D, Beksac M, Spencer A, Oakervee H, Orlowski RZ, Taniwaki M, Röllig C, Einsele H, Wu KL, Singhal A, San-Miguel J, Matsumoto M, Katz J, Bleickardt E, Poulart V, Anderson KC, Richardson P (2015). Elotuzumab therapy for relapsed or refractory multiple myeloma. N Engl J Med.

[24] Maeurer MJ, Gollin SM, Martin D, Swaney W, Bryant J, Castelli C, Robbins P, Parmiani G, Storkus WJ, Lotze MT (1996). Tumor escape from immune recognition: lethal recurrent melanoma in a patient associated with downregulation of the peptide transporter protein TAP-1 and loss of expression of the immunodominant MART-1/Melan-A antigen. J Clin Invest.

[25] Nijhof IS, Casneuf T, Van Velzen J (2016). CD38 expression and complement inhibitors affect response and resistance to daratumumab therapy in myeloma. Blood.

[26] Oka S, Ono K, Nohgawa M (2020). Successful retreatment with elotuzumab for multiple myeloma with extramedullary relapse while being treated with lenalidomide and dexamethasone. Pathol Oncol Res.

[27] Palumbo A, Avet-Loiseau H, Oliva S, Lokhorst HM, Goldschmidt H, Rosinol L, Richardson P, Caltagirone S, Lahuerta JJ, Facon T, Bringhen S, Gay F, Attal M, Passera R, Spencer A, Offidani M, Kumar S, Musto P, Lonial S, Petrucci MT, Orlowski RZ, Zamagni E, Morgan G, Dimopoulos MA, Durie BGM, Anderson KC, Sonneveld P, San Miguel J, Cavo M, Rajkumar SV, Moreau P (2015). Revised international staging system for multiple myeloma: a report from International Myeloma Working Group. J Clin Oncol.

[28] Perez-Simon JA, Sureda A, Fernandez-Aviles F (2006). Reduced-intensity conditioning allogeneic transplantation is associated with a high incidence of extramedullary relapses in multiple myeloma patients. Leukemia.

[29] Raje N, Berdeja J, Lin Y, Siegel D, Jagannath S, Madduri D, Liedtke M, Rosenblatt J, Maus MV, Turka A, Lam LP, Morgan RA, Friedman K, Massaro M, Wang J, Russotti G, Yang Z, Campbell T, Hege K, Petrocca F, Quigley MT, Munshi N, Kochenderfer JN (2019). Anti-BCMA CAR T-cell therapy bb2121 in relapsed or refractory multiple myeloma. N Engl J Med.

[30] Rajkumar SV, Harousseau JL, Durie B, Anderson KC, Dimopoulos M, Kyle R, Blade J, Richardson P, Orlowski R, Siegel D, Jagannath S, Facon T, Avet-Loiseau H, Lonial S, Palumbo A, Zonder J, Ludwig H, Vesole D, Sezer O, Munshi NC, San Miguel J, on behalf of the International Myeloma Workshop Consensus Panel 1 (2011). Consensus recommendations for the uniform reporting of clinical trials: report of the International Myeloma Workshop Consensus Panel 1. Blood.

[31] Rasche L, Bernard C, Topp MS, Kapp M, Duell J, Wesemeier C, Haralambieva E, Maeder U, Einsele H, Knop S (2012). Features of extramedullary myeloma relapse: high proliferation, minimal marrow involvement, adverse cytogenetics: a retrospective single-center study of 24 cases. Ann Hematol.

[32] Rasche L, Rollig C, Stuhler G (2016). Allogeneic hematopoietic cell transplantation in multiple myeloma: focus on longitudinal assessment of donor chimerism, extramedullary disease, and high-risk cytogenetic features. Biol Blood Marrow Transplant.

[33] Rodriguez-Lobato LG, Ganzetti M, Fernandez De Larrea C (2020). CAR T-cells in multiple myeloma: state of the art and future directions. Front Oncol.

[34] Sevcikova S, Minarik J, Stork M, Jelinek T, Pour L, Hajek R (2019). Extramedullary disease in multiple myeloma-controversies and future directions. Blood Rev.

[35] Smith EL, Mailankody S, Staehr M, Wang X, Senechal B, Purdon TJ, Daniyan AF, Geyer MB, Goldberg AD, Mead E, Santomasso BD, Landa J, Rimner A, Riviere I, Landgren O, Brentjens RJ (2019). BCMA-targeted CAR T-cell therapy plus radiotherapy for the treatment of refractory myeloma reveals potential synergy. Cancer Immunol Res.

[36] Soh KT, Tario JD, Jr., Hahn T et al. (2020) CD319 (SLAMF7) an alternative marker for detecting plasma cells in the presence of daratumumab or elotuzumab. Cytometry B Clin Cytom. Online ahead of print 10.1002/cyto.b.21961 PMC8940539 33017079

[37] Spaapen RM, Groen RW, Van Den Oudenalder K (2010). Eradication of medullary multiple myeloma by CD4+ cytotoxic human T lymphocytes directed at a single minor histocompatibility antigen. Clin Cancer Res.

[38] Tai YT, Dillon M, Song W, Leiba M, Li XF, Burger P, Lee AI, Podar K, Hideshima T, Rice AG, van Abbema A, Jesaitis L, Caras I, Law D, Weller E, Xie W, Richardson P, Munshi NC, Mathiot C, Avet-Loiseau H, Afar DEH, Anderson KC (2008). Anti-CS1 humanized monoclonal antibody HuLuc63 inhibits myeloma cell adhesion and induces antibody-dependent cellular cytotoxicity in the bone marrow milieu. Blood.

[39] Usmani SZ, Weiss BM, Plesner T, Bahlis NJ, Belch A, Lonial S, Lokhorst HM, Voorhees PM, Richardson PG, Chari A, Sasser AK, Axel A, Feng H, Uhlar CM, Wang J, Khan I, Ahmadi T, Nahi H (2016). Clinical efficacy of daratumumab monotherapy in patients with heavily pretreated relapsed or refractory multiple myeloma. Blood.

[40] Varga C, Xie W, Laubach J, Ghobrial IM, O'Donnell EK, Weinstock M, Paba-Prada C, Warren D, Maglio ME, Schlossman R, Munshi NC, Raje N, Weller E, Anderson KC, Mitsiades CS, Richardson PG (2015). Development of extramedullary myeloma in the era of novel agents: no evidence of increased risk with lenalidomide-bortezomib combinations. Br J Haematol.

[41] Vij R, Nath R, Afar DEH, Mateos MV, Berdeja JG, Raab MS, Guenther A, Martínez-López J, Jakubowiak AJ, Leleu X, Weisel K, Wong S, Gulbranson S, Sheridan JP, Reddy A, Paiva B, Singhal A, San-Miguel JF, Moreau P (2020). First-in-human phase I study of ABBV-838, an antibody-drug conjugate targeting SLAMF7/CS1 in patients with relapsed and refractory multiple myeloma. Clin Cancer Res.

[42] Zhou X, Fluchter P, Nickel K et al (2020) Carfilzomib based treatment strategies in the management of relapsed/refractory multiple myeloma with extramedullary disease. Cancers (Basel) 12:1035 10.3390/cancers12041035 PMC7225914 32340174

